# Mechanical Properties and Damage Layer Thickness of Green Concrete under a Low-Temperature Environment

**DOI:** 10.3390/ma15217409

**Published:** 2022-10-22

**Authors:** Dongsheng Zhang, Tianhao Zhang, Qiuning Yang

**Affiliations:** 1 School of Civil and Hydraulic Engineering, Ningxia University, Yinchuan 750021, China; 2 Research Group RecyCon, Department of Civil Engineering, KU Leuven, Campus Bruges, 8200 Bruges, Belgium

**Keywords:** low temperature, fly ash, slag, mechanical properties, thickness of damage layer

## Abstract

To study the influence of mineral admixtures on concrete’s mechanical properties after a low-temperature exposure, green concrete was prepared by mixing fly ash and slag at different replacement rates. By analysing the changes to concrete’s mechanical properties and the damage layer thickness under different ambient temperatures (20, −10, −20, −30, and −40 °C), the change rule of concrete at low temperatures was explored. The results revealed that the compressive strength of concrete, containing either fly ash or slag, peaked at 30 °C; moreover, the slag concrete’s flexural and splitting tensile strength peaked at −40 °C. The best mechanical properties were observed for a fly ash-to-slag ratio of 1:2 (F10S20; i.e., 10% fly ash and 20% slag) and its compressive strength at different temperatures was higher than that of concrete, containing 30% fly ash (F30) or 30% slag (S30), but the flexural and splitting tensile strength was lower than S30. Further, as the temperature decreased, the fly ash concrete’s damaged layer thickness gradually increased. When the content of fly ash and slag were both 15% (F15S15), the damaged layer thickness was minimal at different low temperatures, especially at −30 °C, where the thickness was only 8.31 mm.

## 1. Introduction

Concrete, as a primary building material, is widely used in construction projects, bridges, tunnels, hydraulic engineering projects, and other engineering fields. With the emergence of the construction industry in China, the proportion of constructed areas in winter is notably rising. According to incomplete statistics, the construction areas in winter currently account for 30–40% of the total constructed areas [1]. Considering the influence of the global extreme climate with the continuous construction of low-temperature projects in low-temperature zones, such as high-altitude highways, the performance change of concrete in low-temperature environments has received increasing attention. In addition to the northern cold regions, the central and southern parts of China also exhibit occasional extreme temperatures, from −20 °C to −40 °C, during winter [2]. 

The performance of concrete at room temperature and at a high temperature has been fully explored in previous research [3,4,5,6,7]. However, the research on the performance at low temperatures was mainly focused on the period between the 1950s and 1970s. In recent years, the research on low-temperature environments is specifically focused on the aspect of freezing and thawing. The internal structure, water content, and the structure of water corresponding to concrete specimens exposed to low temperatures is subject to change, which is mainly reflected in the mechanical strength of concrete. Research [8] indicates that the compressive strength of concrete at a low temperature or an ultra-low temperature is much higher than that at a normal temperature; however, the change law is extremely complex.

Dahmani [9] discovered that the compressive strength of concrete increases as the temperature decreases. Under the condition of a high water content, the compressive strength of concrete at a low temperature attains a value 2–3 times that of the strength at room temperature. As the temperature decreases, the pore water in the concrete freezes and expands to fill its pores and cracks, thereby enhancing the compactness of the concrete and improving its compressive strength. Therefore, the water content of concrete decisively influences the growth of its compressive strength in a low-temperature environment. Through ultra-low temperature testing, Shan [10] revealed that, as the temperature decreases, the greater the water content, the more significant will be the strength of concrete. Because the free water in the concrete is frozen, the compressive strength of the concrete increases gradually with the reduction of the frozen void. MacLean et al. [11] manufactured cylindrical concrete specimens (27 mm, 101.6 mm × 203.2 mm) and studied the impact on the compressive strength of concrete under simulated extreme climates. A temperature range of 20 °C to −70 °C was selected to obtain the relationship between the temperature and the compressive strength, and the stress–strain relationship curve of the compressive strength was plotted. The compressive strain of the cylindrical concrete specimen was obtained via three-dimensional digital images. Miura [12] and Okada [13] proposed that the relationship between the increment of the concrete’s compressive strength and the temperature is a quadratic power in the range of 0–120 °C. Furthermore, Luis [14] suggested that from −196 °C to 20 °C, the compressive strength of concrete gradually increases as the temperature decreases, and the compressive strength of concrete gradually increases as the concrete’s moisture content increases. Taber [15] has studied this phenomenon for a considerable period and, based on many data and theories, concluded that the frost damage mechanism of concrete in early and late stages is remarkably different; its early damage mechanism is identical to that of frost heave in soil. In the early frost damage, during the freezing process of free water, the pressure difference between the gel pores causes the migration of water, thereby leading to the rapid growth of ice crystals and the generation of pressure. Concurrently, due to the migration and redistribution of water, the internal stress of concrete is redistributed, causing damage to the internal structure of concrete, while the later-stage damage is caused by the volume expansion of water during the freezing process.

The pore volume of concrete expands, owing to the freezing of water [16,17]. Jiang [18] suggested that cement-based materials are heterogeneous, and the thermal stress generated during the temperature reduction acts on their interior structure, which is manifested as the damage to the internal structure of concrete; because of the phase change and the migration of pore water in concrete, the temperature deformation is caused, thereby resulting in the development of the structural deformation through shrinkage and expansion. As reported in the literature [19,20,21], at a low temperature, with the cooling of concrete, the water in the larger pores inside the specimen freezes and expands in volume, and when the tensile stress generated in the skeleton around the pores exceeds the tensile strength of the concrete, the interior of the specimen will crack, and the cracks may appear at the aggregate–slurry interface, the aggregate interface, or the slurry interface. Evidently, when the concrete structure is damaged by freezing, the low temperature seeps into the concrete from the outside to the inside, which gradually freezes and expands, thereby causing concrete damage. The surface damage of the structure is maximally severe, and the inwardly extended damage gradually weakens. Notably, this phenomenon can reflect the characteristics of concrete freezing damage at the macro level [22,23]. In this perspective, the thickness of the damaged concrete layer can optimally describe this feature. With the development of non-destructive testing technology, ultrasonic testing has been increasingly applied for the study of the damaged concrete layer’s thickness. For example, Yuan [24] investigated the change of the damaged concrete layer’s thickness under the action of different concentrations of the sulphate solution, based on the ultrasonic flat measurement method, indicating that this layer’s thickness can reflect the degree of deterioration corresponding to the concrete damage under the action of salt freezing. Chu [25] studied the thickness of the damaged concrete layer with different admixtures under a sulphate attack by using ultrasonic technology, and discovered that the performance of concrete with fly ash is improved over that with slag. Moreover, Ould [26] used ultrasonic testing technology to study the degree of the chemical corrosion of concrete, thereby validating the feasibility of the non-destructive testing of the corrosion degree of concrete with this method. The research on the early freezing of concrete has been delayed, and a systematic and perfect theoretical system is yet to be developed in this field. Moreover, the quantitative research on the early freezing-induced surface damage of concrete, characterized via a non-destructive testing technology is rare. Therefore, simulating the actual engineering environment, using ultrasonic non-destructive testing technology to study the change rule of the thickness of the late damage layer of the frozen concrete, and analysing the damage degree of the early frozen concrete from the perspective of the concrete damage layer are strategies that carry a theoretical significance for studying the durability of the early frozen concrete structures.

Cement generates a large amount of CO_2_ during the production process; to reduce cement consumption, adding mineral admixtures will improve the density of the test pieces and improve the utilisation of industrial solid wastes. Therefore, to investigate the performance changes of concrete at the ambient temperatures of 20 °C, −10 °C, −20 °C, −30 °C, and −40 °C, fly ash and slag are used as the mineral admixtures to partially replace the cement through single and multiple admixtures to make green concrete. By analysing the mechanical properties of the green concrete specimens at a low temperature, including the compressive strength, flexural strength, and the splitting tensile strength, and by measuring the thickness of the damaged layer of the specimens at ultrasonic testing points, the mechanical properties and deterioration damage of the concrete with different mineral admixtures at a low temperature are characterised.

## 2. Materials and Methods

### 2.1. Materials

The cement used in this study is ordinary Portland cement (OPC; Ningxia Saima, Yinchuan, China, PO42.5), and the fineness (retained on a 45 μm sieve) is 3.6%. The coarse aggregate was continuously graded with natural crushed stone (520 mm). The fine aggregate was river sand, with a fineness modulus of 2.71, and the water used was laboratory tap water. The mineral admixture was fly ash and S95 slag, with a fineness (retained on 45 μm sieve) of 23.7% and 4.7%, respectively. The water reducer used is polycarboxylic acid super-plasticiser. The chemical compositions of the cement, fly ash, and slag were tested via X-ray fluorescence spectroscopy (XRF). The results are summarised in Table 1.

### 2.2. Design of Mix Ratio

To explore the influence of the mineral admixtures on the performance of the concrete after exposure to la ow temperature, ordinary concrete was considered the benchmark group, which was recorded as OPC, and fly ash and slag were separately employed to partially replace the cement through single and compound admixtures, of which the single substitution rate of the fly ash and slag was 15, 30, 45, and 60%. The concrete with the single fly ash was recorded as F15, F30, F45, and F60, and the concrete with the single slag was recorded as S15, S30, S45, and S60, respectively. For the concrete mixed with fly ash and slag, the replacement rate of the compound mixing is 30%, in which the proportions of fly ash and slag are 2:1, 1:1, and 1:2, respectively, which were recorded as F20S10, F15S15, and F10S20, respectively. The detailed mix proportions are listed in Table 2.

#### Preparation and Curing of the Test Pieces

First, the cementing material and river sand were poured into the concrete mixing pot and mixed for 30 s. Subsequently, water and the water reducing agent were added and mixed for 60 s to yield a uniformly distributed mixture. Finally, the coarse bone aggregate was added and mixed for 90 s, and thereafter, the mixture was discharged and installed into the mould. Following the vibrating and moulding, the mixture was cured at room temperature for 24 h. Subsequently, the mould was removed, and the set mixture was placed into a standard curing room. Following the curing of the mixture, for 28 d under standard conditions (20 ± 2 °C; relative humidity: 95%), it was retrieved from the room. The surface was allowed to completely dry, and a low-temperature test was conducted thereafter.

### 2.3. Experimental Methods

#### 2.3.1. Low-Temperature Test

**Figure 1:**
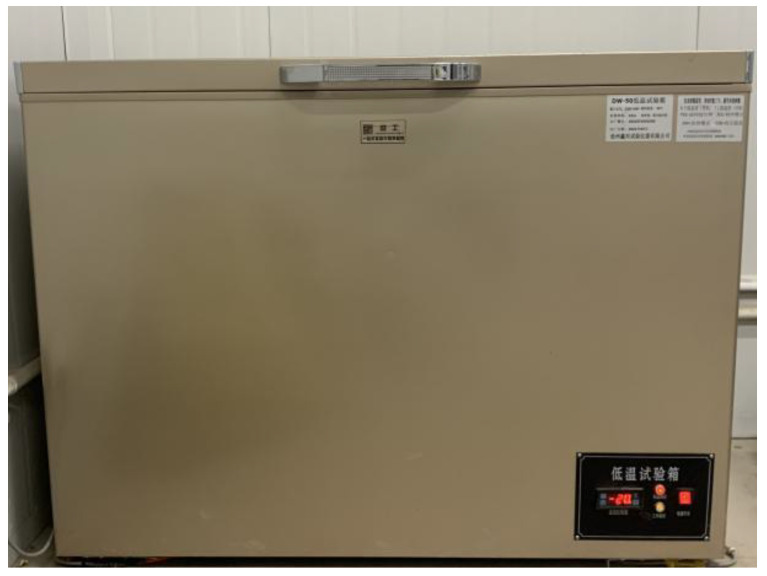
Low-temperature test chamber.

The DW-50 low-temperature box, produced by the China Cangzhou Xinxing Experimental Instrument Co. Ltd., is used to freeze the concrete at a low temperature, as shown in Figure 1. The test piece with a dry surface was gently placed in the low-temperature chamber to freeze. To ensure that the concrete test piece is fully frozen at the ambient temperature, the temperature was kept constant for 4 h after the internal temperature of the low-temperature chamber reached the target temperature (−10, −20, −30, or −40 °C). Thereafter, the test pieces were retrieved and measured to determine their mechanical properties, and the thickness of the damaged layer.

#### 2.3.2. Mechanical Properties

The mechanical property test refers to BS EN 12390-4 (2000) [27], in which 100 mm × 100 mm × 100 mm cubes are used for determining the compressive and splitting strength, and a 100 mm × 100 mm × 400 mm prism test piece is used to measure the bending strength. Three test pieces are considered a group, and the average value is measured after the test.

#### 2.3.3. Damaged Layer Detection

**Figure 2:**
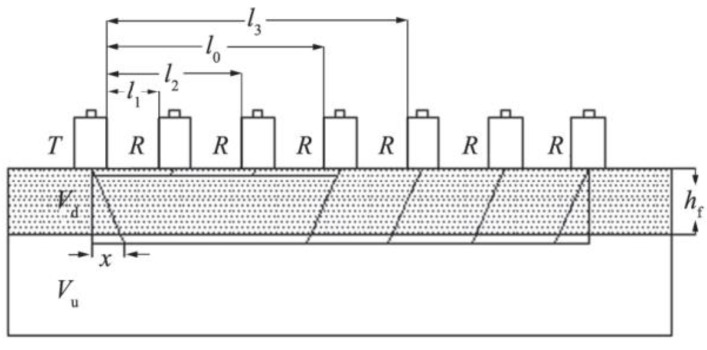
Schematic illustration of the horizontal survey [29].

The ultrasonic testing of the concrete surface’s damaged layer is an indirect testing method. As per the basic principle of this method, sound waves travel at different speeds in the media with different densities, and the refraction and reflection occur when they pass through the interface of the media with different densities. When the concrete structure experiences early frost damage, the low temperature will gradually penetrate the concrete from the outside to the inside, which invariably causes varying degrees of damage on the concrete surface, thereby loosening the surface concrete and yielding damage layers of different thicknesses. For a large thickness of the concrete’s damaged layer, this parameter will affect the durability and bearing capacity of the concrete, so the ultrasonic test is an important parametric index in the concrete non-destructive testing technology [28]. In accordance with the single plane measurement method reported in the literature [29], the side of the prism test piece was tested. The installation method is illustrated in Figure 2. The transmitting transducer is placed 50 mm away from the edge, and the receiving transducer (R) is placed along the 100 mm × 400 mm side and equipped with 50, 75, 100, 150, 200, 250, and 300 mm ranging devices for troubleshooting. Finally, the sound time values of the different ranging devices are read. Vaseline is used as the coupling agent. The thickness of the damaged layer can be calculated using the following formula:

(1) $$l_{0}=\frac{{(a}_{1}b_{2}-a_{2}b_{1})}{{(b}_{2}-b_{1})}$$

(2) $$h_{f}=\frac{l_{0}}{2}\cdot \sqrt{\frac{{(b}_{2}-b_{1})}{{(b}_{2}{+b}_{1})}}$$

where, *h_f_* is the thickness of the damaged layer (mm); ${\;a}_{1},{\;a}_{2\;},{\;b}_{1\;}{\;\mathrm{and}\;b}_{2}\;$ are the regression coefficients.

## 3. Results

### 3.1. Compressive Strength

**Figure 3:**
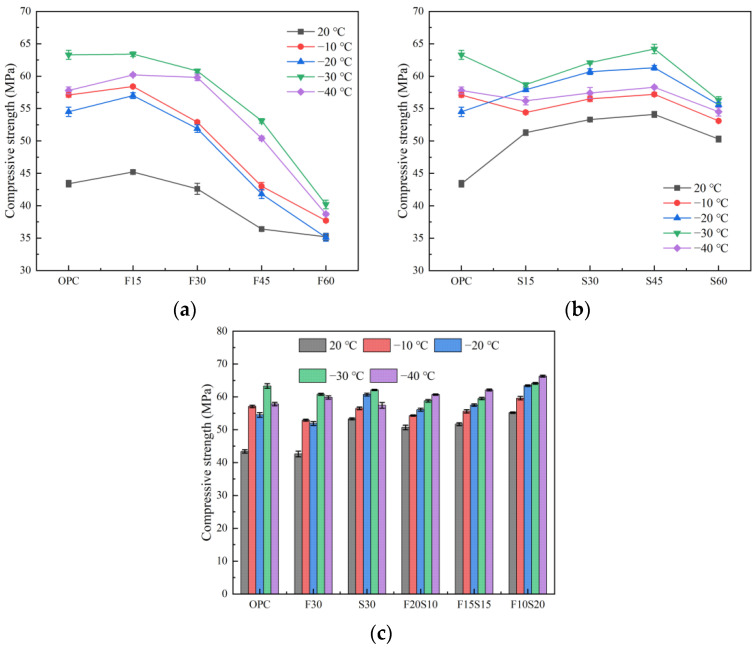
Compressive strength of the concrete. (**a**) Mixed with fly ash, (**b**) mixed with slag, and (**c**) mixed with fly ash and slag.

Figure 3a depicts the schematic illustration of the results corresponding to the compressive strength test of the test pieces. The strength of the specimen decreases with the increase in the content of fly ash, and the strength of the specimen with 15% fly ash is the highest. This remarkable strength is a result of the fly ash’s microaggregate filling effect in addition to the pozzolanic activity [30,31]. The appropriate amount of fly ash fills the pores partially, thereby improving the compactness of the concrete and the strength of the concrete. When the content of fly ash is 60%, the strength of the specimen is the lowest at all temperatures. At −20 °C to −40 °C, the strength of the F60 group decreases by 18.9, 34.0, 35.6, 37.1, and 33.1%, respectively, compared with the OPC. Notably, the influence of the temperature on the compressive strength of the fly ash concrete is evident. The strength of the fly ash concrete after a low-temperature exposure is higher than that under a normal temperature. The strength is most significantly improved at −30 °C; those for the OPC, F15, F30, F45, and F60, increased from 43.4, 45.2, 42.6, 36.4, and 35.2 MPa, under a normal temperature to 63.9, 63.4, 60.8, 53.1, and 40.2 MPa, respectively, followed by those at −40, −10, and −20 °C. This is because the compressive strength of the concrete at a low temperature mainly depends on the water content. As the temperature decreases, the water in the concrete pores will condense into ice to fill the pores. Simultaneously, the bonding strength between the hydrophilic silicate materials increases, which increases the compressive strength of the concrete [32,33,34].

Figure 3b illustrates the schematic illustration of the compressive strength under the condition of a single slag mixing. Observably, the strength of the test piece initially increases and subsequently decreases with the increase of the slag content. When the slag content is 45%, the strength attains its maximum value under the various temperature conditions, and a content of 60% slag renders the compressive strength of S60 slightly lower than that of the OPC. Similarly, the strength of the slag concrete after a low-temperature exposure is higher than that under a normal temperature, and especially at −30 °C, the strength is increased most significantly, followed by the improvements at −20, −40, and−10 °C.

The results plotted in Figure 3c depict the compressive strength of the test piece under the condition of double mixing. Under the condition of the double mixing of fly ash and slag, the strength of the test piece increases with the increase in the amount of slag. When the fly ash-to-slag ratio is 1:2 (F10S20), i.e., when the slag content is 20% and the fly ash content is 10%, the compressive strength of the test piece attains its maximum value, and the strength under −40 °C reaches the peak value, which is 66.3 MPa. Compared with ordinary concrete, the compressive strength of the F10S20 group is higher than the OPC at other temperatures, except that the strength after −30 °C is slightly lower than that of the ordinary concrete. Compared with the single fly ash and single slag mixing, F20S10 and F15S15 offer no obvious strength advantages after a low-temperature exposure; on the contrary, they even exhibit an insufficient strength at certain temperatures. The compressive strength of the F10S20 group after exposure to different low temperatures is greater than that of F30 and S30, thereby validating that the proper addition of slag can greatly improve the density of the fly ash concrete; this strategy achieves the purpose of improving the strength of the concrete. In addition, in contrast to the single mixing rule, the strength of the concrete under the combined mixing strategy increases with the decrease of strength.

#### 3.1.1. Flexural Strength

**Figure 4:**
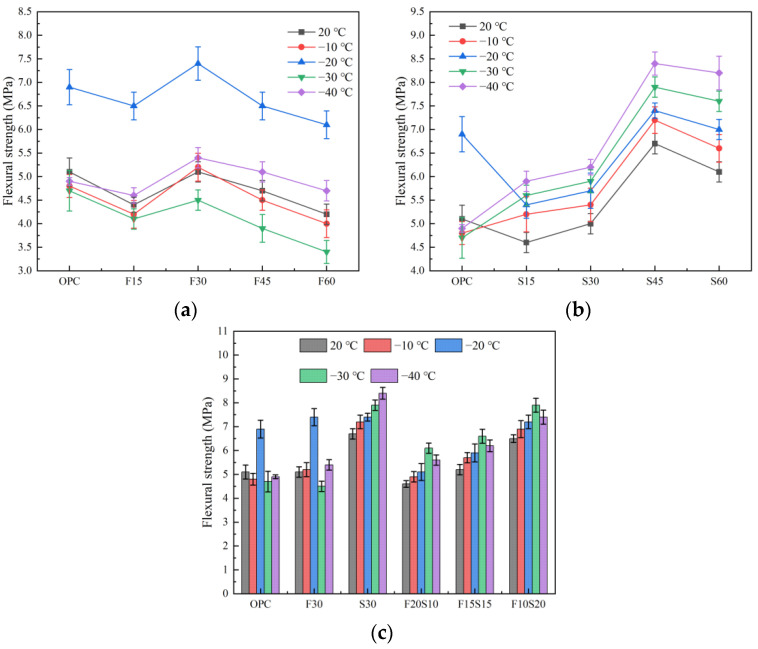
Flexural strength of the concrete. (**a**) Mixed with fly ash, (**b**) mixed with slag, and (**c**) mixed with fly ash and slag.

Figure 4 presents the schematic illustration of the flexural strength results of the tested concrete. As illustrated in Figure 4a, with the increase of the fly ash content, the flexural strength of the concrete first increases and thereafter decreases. Under exposure to different low temperatures, the flexural strength of the F30 group is higher than those of the other groups. Among them, at −20 °C, the flexural strength of the fly-ash concrete is most significantly improved, and the OPC, F15, F30, F45, and F60, are improved from 5.1, 4.4, 5.1, 4.7, and 4.2 MPa, at room temperature to 6.9, 6.5, 7.4, 6.5, and 6.1 MPa, respectively. The flexural strength of the fly ash concrete after exposure to −30 °C, is lower than that of the concrete at room temperature, especially F60, which is only 80.9% at room temperature.

As observed in Figure 4b, the flexural strength of the concrete increases initially and subsequently decreases with the increase of the slag content. When the content is 45%, the flexural strength is the largest, which is consistent with the change rule of the compressive strength. The main difference is that for the OPC, the flexural strength of the concrete at −20 °C is improved, which is 35.3% higher than that at room temperature, especially at −30 °C, the flexural strength of the OPC is maximally damaged. In contrast to that for the OPC, a low-temperature exposure improves the flexural strength of the mineral-powder concrete at different dosages. With the decrease of the temperature, the strength of the concrete increases, because the movement of free water and the damage in the frozen test piece led to a significant reduction in the flexural strength. Moreover, as the temperature continues to decrease, the water in the pores gradually starts to freeze, the pores are filled, and the resistance of the test piece to external forces gradually increases [34,35], thereby leading to a gradual increase in the flexural strength. At −40 °C, the flexural strength of the specimen is maximal. Currently, the flexural strength of the S45 group reaches 8.4 MPa, which is 25.4% higher than that at a normal temperature, 71.4% higher than that of the OPC group at the same temperature, and 64.7% higher than that of the fly ash specimen (F45) at the same dosage and temperature.

Figure 4c depicts the compressive strength of the test piece under the condition of double mixing. Observably, under the condition of the double mixing of fly ash and slag, the strength of the test piece increases with the increase in the amount of slag. When the fly ash-to-slag ratio is 1:2, the flexural strength of the F10S20 group attains its maximum value, and −30 °C is the optimal temperature for the compound mixing. At this temperature, the flexural strength of the F10S20 group is 7.9 MPa, followed by those at −40, −20, and −10 °C. The evident difference from the change rule of the compressive strength is that the F20S10 and F15S15 groups do not offer obvious strength advantages after a low-temperature exposure compared with the single fly ash (F30), and even the strength at certain temperatures is insufficient. However, regardless of the proportion in which the fly ash and slag is mixed, the flexural strength of the concrete at different low temperatures is lower than that of the concrete with only slag (S30). Accordingly, the effect of the mixed fly ash and slag on the flexural strength of the concrete after a low-temperature exposure, is not as desirable compared to that of the concrete with only slag.

#### 3.1.2. Splitting Tensile Strength

**Figure 5:**
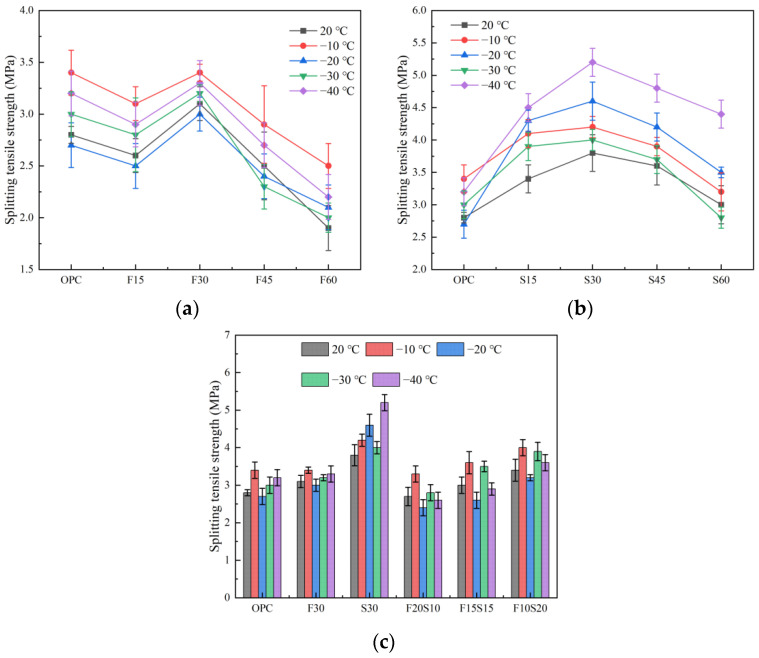
Splitting tensile strength of the concrete. (**a**) Mixed with fly ash, (**b**) mixed with slag, and (**c**) mixed with fly ash and slag.

Figure 5 represents the schematic illustration of the results of the splitting tensile strength of the test pieces. As demonstrated by the plot in Figure 5a, the splitting tensile strength of the concrete initially increases and subsequently decreases with the increase in the amount of fly ash. In addition, the splitting tensile strength of the fly ash concrete at −10 °C after exposure to a low temperature, increases considerably, and the strength of the F30 group attains the peak value of 3.4 MPa at −10 °C. Compared with the OPC, except for the F30 group, the splitting tensile strength of other fly ash concrete specimens at a low temperature is lower than that of the OPC, especially for the F60 group.

For the single-slag specimen, the splitting tensile strength reaches its peak value at 30%. At this time, the exposure to −40 °C exerts the most significant effect on the splitting tensile strength of the slag concrete, followed by the effects at −20, −10, and −30 °C. Excluding the development that the splitting tensile strength of the S60 group after exposure to −30 °C is slightly lower than that of the OPC, the splitting tensile strength of the slag concrete after exposure to different low temperatures, is higher than that of the OPC, indicating that the addition of slag has a positive effect on the splitting tensile strength of concrete.

The splitting tensile strength of the concrete mixed with fly ash and slag is illustrated in Figure 5c. The bending strength of the specimen increases with the increase of the slag content, but when the ratio of the fly ash-to-slag is 2:1, the splitting tensile strength of the F20S10 group at different temperatures is lower than that of the OPC, F30, and S30. When the slag content reaches 20%, although the splitting tensile strength of F10S20 after a low-temperature treatment is improved, rendering it greater than that of the OPC and F30, its strength is still lower than that of S30. The splitting tensile strength at 20, −10, −20, −30, and −40 °C is only 89.5, 95.2, 69.6, 97.5, and 69.2% of S30, respectively. In addition, regardless of the proportion of the fly ash and slag used, −10 °C exerted the most obvious effect on the splitting tensile strength of the concrete, followed by the improvements offered by −30, −40, and −10 °C.

### 3.2. Damage Layer Thickness 

**Figure 6:**
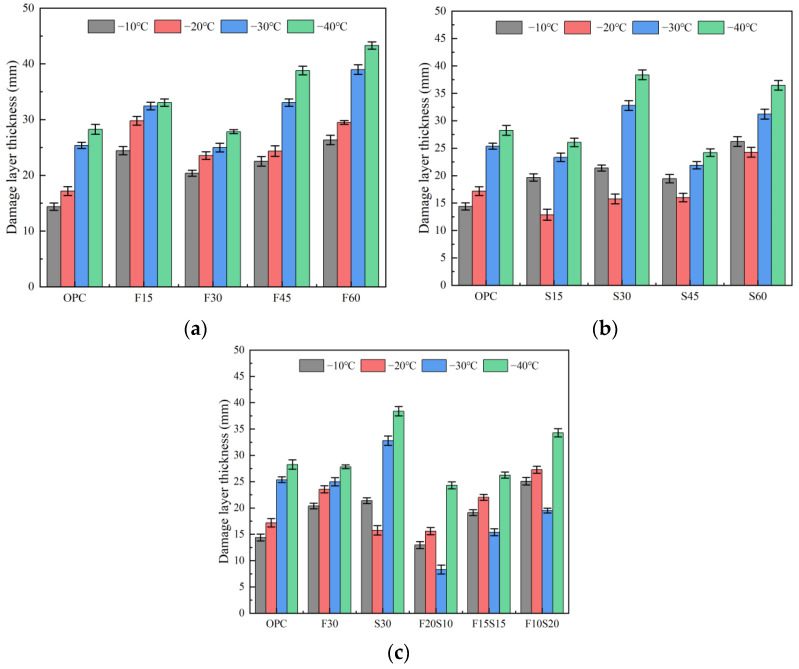
Thickness of the damaged layer of the concrete. When mixed with (**a**) fly ash, (**b**) slag, and (**c**) fly ash and slag.

The thickness of the damaged layer of each group of test pieces at different temperatures is depicted in Figure 6. The thickness of the damaged layer of each group of the test pieces increases with the increase in the content of the supplementary cementitious materials. As indicated in Figure 6a and Table 2, the thickness of the damaged layer of both the OPC and fly ash single-mixed specimens increases with the decrease of temperature, and the damage thickness of the OPC at −40 °C is 28.25 mm. In addition, with the increase of the fly ash content, the damaged layer’s thickness of the concrete increases accordingly. When the fly ash content is 60% and the ambient temperature attains a value of −40 °C, the maximum thickness of the damaged layer of F60 reaches 43.30 mm, which is 53.5, 31.1, 55.6, and 11.6% higher than that of the OPC, F15, F30, and F45, respectively. This trend may be a result of the propagation speed of the ultrasonic wave in the concrete, which is related to the compactness of the concrete [35]. When the amount of fly ash is extremely large, the compactness of the slurry is inadequate, owing to its low activity and its inability to fully hydrate into gel, thereby reducing the self-compacting concrete. With the secondary hydration of the fly ash, a large amount of Ca(OH)_2_ is consumed, greatly reducing the compactness of the test pieces [36,37,38]. The thickness of the damaged layer increases with the decrease in temperature because the free water in the test piece moves continuously and forcefully in the direction of the high temperature. The decrease in temperature leads to the enlargement of the pores in the test piece and the appearance of cracks; thus, the compactness of the concrete decreases further, eventually leading to the increase of the damaged layer of the test piece.

As illustrated in Figure 6b, the thickness of the damaged layer of the test piece increases with the increase in the amount of slag added under the condition of the single slag addition. This is because, under low-temperature conditions, the temperature difference between the inside and outside of the concrete is substantial, which leads to the generation of thermal stress inside the concrete. During thermal stress, the concrete interior will be damaged, which leads to an increase in the damage layer thickness of the specimen [39]. Concurrently, the damaged layer’s thickness initially decreases and subsequently increases with the decrease in temperature. At the same dosage, the thickness of the damaged layer of the concrete specimen with the single slag is less than that of the concrete specimen with the single fly ash. The reason for this phenomenon may be that the activity of slag is relatively strong, which will increase the degree of hydration of the test piece after mixing, and thus, the hydration products will increase, improving its compactness. The reduction of the ambient temperature will lead to the internal cracking of the test piece. However, under the initial conditions, the compactness of the test piece obtained solely with slag is acceptable; thus, the thickness of the damaged layer is lower than that of the test piece with the fly ash alone.

The change rule of the thickness of the damaged concrete layer caused by the fly ash and slag is illustrated in Figure 6c. With the increase in the amount of slag, the thickness of the damaged concrete layer at a low temperature initially decreases and subsequently increases. For the fly ash: slag = 1:1 (group F15S15), the thickness of the damaged layer is optimal and lower than that of the OPC, F30, and S30. When the temperature drops from the normal temperature (20 °C) to −20 °C, the thickness of the damaged layer of the concrete increases. The thickness of the damaged layer of F15S15 at −20 °C is 15.6 mm, which is 90.8, 66.3, 99.1, 70.9, and 57.2% of the OPC, F30, S30, F20S10, and F10S20, respectively. With the continuous decrease in temperature, the thickness of the damaged layer of the concrete at −30 °C decreases and reaches its minimum value, which is only 8.31 mm and 67.3, 66.7, 74.7, 46, and 57.4% lower than that of the OPC, F30, S30, F20S10, and F10S20, respectively. When the ambient temperature drops to –40 °C, the thickness of the damaged layer increases to 24.31 mm, which is 65.8% higher than that at −30 °C, and it is 86.1, 87.4, 63.3, 92.6, and 71% that of the OPC, F30, S30, F20S10 and F10S20.

## 4. Conclusions

(1) As the fly ash content was increased (1560%), the compressive strength of the concrete gradually decreased, and the strength of the concrete (F15) was maximal when the content was 15%. The flexural strength and the splitting tensile strength of the concrete increased first and then decreased as the fly ash content was increased, and the strength was maximal when the fly ash content was 30% (F30). With the decrease in temperature, the strength of the fly ash concrete fluctuated greatly. In terms of the compressive strength, the strength of fly-ash concrete at different low temperatures was higher than that of the concrete at room temperature, especially at −30 °C, followed by those at −40, −10, and −20 °C. In terms of the flexural strength, the strength of the fly ash concrete at −20 °C was significantly higher than that at other temperatures. For the −40 ℃ variant, the flexural strength and splitting tensile strength attained the maximum peak.
(2) The positive influence of the slag on the mechanical properties of the concrete was considerable. When the slag amount was 30% (S30) within the test range, the splitting tensile strength was maximal, and when the slag amount was 45% (S45), the compressive strength and flexural strength of the concrete were the highest. Slag concrete, exposed to a low temperature of −30 °C, yielded the maximum compressive strength; for the −40 °C variant, the flexural strength and splitting tensile strength attained the maximum peak.
(3) Under the condition of the double mixing of the fly ash and slag, the concrete strength at room temperature was greatly improved, compared with the OPC due to the synergistic effect of the fly ash and slag. When fly ash: slag = 2:1 (F10S20), the mechanical properties are superior to the rest, and the compressive strength is maximal, which is 127.2, 129.6, and 103.6% of the OPC, F30 and S30, respectively. The compressive strength at different temperatures was higher than that of the F30 and S30 groups, and the optimum temperature was −40 °C. However, its flexural strength at different temperatures was lower than that of the S30 group, but the flexural strength at other temperatures was higher than that of F30 group, except at −20 °C. Notably, its splitting strength is less than that of the S30 group at different temperatures.
(4) During low temperatures, the thermal stress will be generated in the concrete. During thermal stress, the damage layer thickness of the concrete will increase as the temperature decreases. For the single fly ash blending, the maximum value of the damage layer thickness is 43.3 mm at −40 °C. As the slag content increases, the damage layer thickness first decreases and then increases, and as the temperature decreases, the damage layer thickness of the concrete increases. For the concrete specimen containing both the fly ash and slag, when the fly ash-to-slag ratio is 1:1 (F15S15), the damage layer thickness is the smallest, and is superior to the OPC, F30, and S30. At −30 °C, the damage layer thickness of the concrete is 8.31 mm, which is 32.8%, 33.3%, and 25.3% of the OPC, F30 and S30, respectively.

This paper mainly focused on the influence of different temperatures on the macro-performance of concrete under low-temperature conditions. However, factors, such as the pore distribution, the structure of concrete at low temperatures, and the relationship between the pore size and the freezing point will affect the mechanical properties and damage of concrete at low temperatures. Therefore, in future research, it is necessary to further explore the micro-morphology and mechanism of the concrete at low temperatures.

## Figures and Tables

**Table 1:** Chemical properties of the cement, fly ash, and slag (%).

Oxide	Al_2_O_3_	SiO_2_	Fe_2_O_3_	CaO	Na_2_O	K_2_O	SO_3_
Cement	7.60	22.46	5.00	57.15	0.31	0.86	2.96
Fly ash	26.38	47.85	8.43	5.81	1.11	2.22	1.94
Slag	14.8	28.7	0.43	38.1	1.78	0.66	2.18

**Table 2:** Proportions of the different mixtures (kg/m^3^).

Code	Fly Ash	Slag	Cement	Water	Sand	Coarse Aggregate	Super-Plasticiser
OPC	0	0	400	160	756	1134	3
F15	60	0	340				4.3
F30	120	0	280				5.2
F45	180	0	220				6.1
F60	240	0	160				7.1
S15	0	60	340				3.4
S30	0	120	280				3.9
S45	0	180	220				4.6
S60	0	240	160				5.0
F20S10	80	40	280				4.7
F15S15	60	60	280				4.4
F10S20	40	80	280				3.8

## Data Availability

Not applicable.
